# Regulation of Thrombomodulin Expression and Release in Human Aortic Endothelial Cells by Cyclic Strain

**DOI:** 10.1371/journal.pone.0108254

**Published:** 2014-09-19

**Authors:** Fiona A. Martin, Alisha McLoughlin, Keith D. Rochfort, Colin Davenport, Ronan P. Murphy, Philip M. Cummins

**Affiliations:** 1 School of Biotechnology, Dublin City University, Glasnevin, Dublin, Ireland; 2 School of Health & Human Performance, Dublin City University, Glasnevin, Dublin, Ireland; 3 Centre for Preventive Medicine, Dublin City University, Glasnevin, Dublin, Ireland; Emory University/Georgia Insititute of Technology, United States of America

## Abstract

**Background and Objectives:**

Thrombomodulin (TM), an integral membrane glycoprotein expressed on the lumenal surface of vascular endothelial cells, promotes anti-coagulant and anti-inflammatory properties. Release of functional TM from the endothelium surface into plasma has also been reported. Much is still unknown however about how endothelial TM is regulated by physiologic hemodynamic forces (and particularly cyclic strain) intrinsic to endothelial-mediated vascular homeostasis.

**Methods:**

This study employed human aortic endothelial cells (HAECs) to investigate the effects of equibiaxial cyclic strain (7.5%, 60 cycles/min, 24 hrs), and to a lesser extent, laminar shear stress (10 dynes/cm^2^, 24 hrs), on TM expression and release. Time-, dose- and frequency-dependency studies were performed.

**Results:**

Our initial studies demonstrated that cyclic strain strongly downregulated TM expression in a p38- and receptor tyrosine kinase-dependent manner. This was in contrast to the upregulatory effect of shear stress. Moreover, both forces significantly upregulated TM release over a 48 hr period. With continuing focus on the cyclic strain-induced TM release, we noted both dose (0–7.5%) and frequency (0.5–2.0 Hz) dependency, with no attenuation of strain-induced TM release observed following inhibition of MAP kinases (p38, ERK-1/2), receptor tyrosine kinase, or eNOS. The concerted impact of cyclic strain and inflammatory mediators on TM release from HAECs was also investigated. In this respect, both TNFα (100 ng/ml) and ox-LDL (10–50 µg/ml) appeared to potentiate strain-induced TM release. Finally, inhibition of neither MMPs (GM6001) nor rhomboids (3,4-dichloroisocoumarin) had any effect on strain-induced TM release. However, significantly elevated levels (2.1 fold) of TM were observed in isolated microparticle fractions following 7.5% strain for 24 hrs.

**Conclusions:**

A preliminary *in vitro* investigation into the effects of cyclic strain on TM in HAECs is presented. Physiologic cyclic strain was observed to downregulate TM expression, whilst upregulating in a time-, dose- and frequency-dependent manner the release of TM.

## Introduction

Thrombomodulin (TM), a multi-domain type-1 membrane glycoprotein constitutively expressed on the lumenal surface of vascular endothelial cells, binds circulating thrombin to elicit the concomitant activation of protein C (amongst various other homeostatic actions). As such, TM is a central determinant of vascular endothelial thromboresistance by promoting anti-coagulant and anti-inflammatory properties within the vessel wall [1]. Shedding or release of soluble TM (sTM) into circulating blood has also been widely reported [2]–[5].

Given the importance of TM to vascular homeostasis, a clearer understanding of how it is regulated within the vascular endothelium by physiological hemodynamic forces is of significant interest. Blood flow-associated hemodynamic forces, namely cyclic strain (stretch) and laminar shear stress, within specific physiological limits, typically work in concert to exert a beneficial influence on endothelial-dependent regulation of vessel homeostasis [6]. In this regard, endothelial cells employ well characterised mechanosensor mechanisms to enable them to sense and respond to their hemodynamic environment, thus facilitating either acute or chronic remodeling of blood vessel architecture to complement circulatory conditions [7], [8]. Moreover, dysregulation (e.g. attenuation, hyper-elevation) of either of these forces can contribute to endothelial activation that may lead to vessel remodeling and vascular diseases (e.g. atherosclerosis, hypertension, stroke, vein graft thrombosis, ventilator-induced lung injury, retinopathy) [9]–[13].

Some regulatory links between endothelial TM expression and hemodynamic forces have previously been demonstrated. Shear-dependent up-regulation of TM expression has been reported in human retinal microvascular endothelial cells [14], human umbilical vein endothelial cells (HUVECs) [15], human abdominal aortic endothelial cells (HAAECs) [16], and even in a mouse transverse aortic constriction model of flow-dependent remodeling [17], observations basically consistent with the atheroprotective nature of laminar shear. However, the effect on endothelial TM expression of physiologic cyclic strain, the repetitive outward stretching of the vessel wall in synchronization with the cardiac cycle, has received considerably less attention in the literature. Sperry *et al.* [18] have previously demonstrated strain-dependent reduction in endothelial TM expression within rabbit autologous vein grafts. Interestingly, these *in vivo* observations contrast with those of Chen *et al.* [19] who demonstrated a sustained increase in TM protein expression following 21% cyclic strain of HUVECs, the pathological levels of strain applied in this study unfortunately rendering these observations somewhat difficult to interpret.

With the exception of the aforementioned *ex vivo* study by Sperry and co-workers, to our knowledge there are no existing *in vitro* studies investigating the influence of physiologic cyclic strain on TM expression in vascular endothelial cells, or indeed the influence of either physiologic cyclic strain or shear stress on endothelial TM release. This paper now addresses this knowledge deficit using human aortic endothelial cell (HAEC) culture models. Particular emphasis is placed on how physiologic levels of cyclic strain, the lesser studied force with respect to TM regulation, may influence the expression and release of endothelial TM.

## Materials and Methods

### Materials

Unless otherwise stated, all reagents were purchased from Sigma-Aldrich (Dublin, IRL). All primers were purchased from Eurofins MWG Operon (London, UK).

### Cell culture

Primary-derived human aortic endothelial cells (HAECs) were obtained from Promocell GmBH (Heidelberg, Germany - Cat No. C-12271) and routinely grown in Promocell Endothelial Cell Growth Media MV (Cat No. C-22020) supplemented with 5% fetal calf serum, 0.4% endothelial cell growth supplement/bovine hypothalamic extract, heparin (90 µg/mL), hydrocortisone (1 µg/ml), epidermal growth factor (10 ng/mL), and antibiotics (100 U/mL penicillin, 100 µg/mL streptomycin). All cells (passages 5–12) were grown and maintained in a humidified atmosphere of 5% CO_2_/95% air at 37°C.

### Hemodynamic force studies

For cyclic strain (CS) studies, the earlier method of Sweeney *et al.* was employed with minor modifications [20]. HAECs were seeded into 6-well ProNectin-coated Bioflex plates (Dunn Labortechnik GmBH - Asbach, Germany) at a density of approximately 5×10^5^ cells/well. At confluency, a Flexercell Tension Plus FX-4000T system (Flexcell International Corp. – NC, USA) was subsequently used to apply a physiological level of equibiaxial cyclic strain to each plate (0–7.5% strain, 60 cycles/min, 0–48 hr, cardiac waveform). Cells were also seeded into standard 6-well plates and exposed to physiological levels of laminar shear stress (LSS) (10 dynes/cm^2^, 0–48 hr) on an orbital rotator as previously described [21], [22].

Following experiments, both cells and conditioned media were routinely harvested for analysis. For cell lysate preparation, cells were washed thrice in PBS before being scraped into radioimmunoprecipitation assay (RIPA) lysis buffer (64 mM HEPES pH 7.5, 192 mM NaCL, 1.28% w/v Triton X-100, 0.64% w/v sodium deoxycholate, 0.128% w/v sodium dodecyl sulfate, 0.5 M sodium fluoride, 0.5 M EDTA, 0.1 M sodium phosphate, 10 mM sodium orthovanadate, and 1× protease/phosphatase inhibitor cocktail) and transferred into a pre-chilled micro-centrifuge tube. Continuous lysate rotation was applied for 1 hr at 4°C, prior to lysate clarification by centrifugation at 10,000×g for 20 min at 4°C to sediment any triton-insoluble material. Clarified lysates were quantified by BCA assay [23]. Conditioned media samples were collected from each Bioflex well (1 ml/well, 2 ml/well for microparticle fractionation studies) and centrifuged at 700×g for 15 min to remove crude cellular debris. Media samples were routinely assayed by ELISA prior to freezing. All samples were ultimately stored at −80°C.

### Microparticle fractionation and flow cytometry

For the isolation of microparticles present in HAEC-conditioned media, the methods of Lacroix *et al.* were employed with minor modifications [24], [25]. Following experiments, conditioned media was harvested and initially centrifuged at 300×g for 5 min and subsequently at 2500×g for 10 min to facilitate removal of cellular debris. Media was then centrifuged at 20,000×g for 90 min, after which the supernatant was set aside and the pellet resuspended in 1 ml of phosphate buffered saline (PBS). The washed pellet was subjected to a second spin at 20,000×g for 90 min to yield a final microparticle pellet that was resuspended in lysis buffer (100 mM Tris HCl, pH 8.1, 0.5% Triton X-100, protease inhibitor mix). Microparticle lysates were then stored at −80°C until required for TM ELISA. All centrifugation steps proceeded at 4°C.

For analysis of the effect of cyclic strain on endothelial MP production, we employed flow cytometry of non-lysed MP fractions (isolated as described above) following cyclic strain for 24 hrs at 0 or 7.5%. Briefly, MP fractions were resuspended in 400 µl of FACS buffer (filtered PBS containing 2% fetal bovine serum and 0.1% sodium azide). PE-conjugated anti-VE-cadherin mouse IgG (Catalog 560410, BD Biosciences, Oxford, UK) and FITC-conjugated recombinant annexin V (Catalog 31490013, ImmunoTools, Germany) were added to MP fractions (20 µl and 5 µl, respectively) and left to incubate as per manufacturer guidelines (60 min for VE-cadherin and 15 min for annexin V). Following incubation, the MPs were pelleted and resuspended in a final volume of 250 µl of fresh FACS buffer before being read by flow cytometry for 60 seconds at a fixed flow rate to assess MP levels. The flow cytometer (FACS Aria) was pre-calibrated using a 2% suspension of standard 0.1 µm polystyrene-latex beads (Catalog LB-1, Sigma-Aldrich) with further optimisation performed using a suspension of MPs isolated from control HAECs. Both single- and double-stained MP control samples were employed for compensation purposes. Final values were normalised per 1×10^5^ cells. All FACS data analysis employed FlowJo software.

### Real-time PCR

Following experiments, endothelial cells were harvested for extraction of total RNA according to the method of Chomczynski and Sacchi [26] using TRIzol reagent (Life Technologies, Dublin, IRL). cDNA was generated from 1 µg of total RNA using a high capacity cDNA reverse transcription kit (Life Technologies). Quantitation of the final cDNA was determined using a NanoDrop 3300 Spectrophotometer (Thermo Scientific, DE, USA). Amplification of target cDNA sequences using gene-specific primers was subsequently performed and analyzed as previously described [27]. Ribosomal subunit S18 was routinely used for normalization purposes. Primer pairs (shown below) were screened for correct product size (1% agarose gel electrophoresis) and underwent melt-curve analysis for primer-dimers. TM (107 bp): Forward 5′-ACCTTCCTCAATGCCAGTCAG-3′; Reverse 5′-GCCGTCGCCGTTCAGTAG-3′; S18 (250 bp): Forward 5′-CAGCCACCCGAGATTGAGCA-3′; Reverse 5′- TAGTAGCGACGGGCGGTGTG-3′.

### Western immunoblotting

Western blotting was employed to confirm TM protein expression and for semi-quantitative comparison of cellular TM levels between control and strained samples. Following experiments, endothelial cell lysates were harvested, resolved by 10% SDS-PAGE under reducing conditions, and electroblotted as previously described [27]. Membranes were blocked for 60 min in tris-buffered saline (TBS: 10 mM Tris pH 8.0, 150 mM NaCl) containing 5% w/v skim milk before being incubated overnight in primary antisera with gentle agitation at 4°C. Primary antisera were prepared in TBS (+5% skim milk): 10 µg/ml anti-TM mouse polyclonal IgG (Abcam, Cambridge, UK) and 1 µg/mL anti-GAPDH rabbit monoclonal IgG (Santa Cruz Biotechnology, CA, USA). Membranes were then washed thrice in TBS containing 0.1% Tween (TBST) before being incubated for 2 hrs in secondary antisera with gentle agitation at room temperature. Secondary antisera (Amersham Pharmacia Biotech, Buckinghamshire, UK) were prepared in TBST (+5% milk): 1/3000 HRP-conjugated goat anti-mouse IgG (TM) and 1/3000 HRP-conjugated goat anti-rabbit IgG (GAPDH). Membranes were developed using a Luminata Western HRP kit (Millipore, Cork, IRL) followed by chemiluminescent imaging using a G-Box gel-documentation system (Syngene, UK). Scanning densitometry of Western blots was performed uysing NIH ImageJ software.

### Enzyme-linked immunosorbent assay (ELISA)

A Thrombomodulin/BDCA-3 DuoSet ELISA Kit (R&D Systems, MN, USA) was employed as per manufacturer instructions (with minor modifications) to accurately measure absolute TM levels in HAEC lysates and conditioned media. Briefly, F96 maxisorp Nunc-Immuno 96-well plates (Bio-Sciences Ltd., Dun Laoghaoire, IRL) were coated with 50 µl/well of the capture antibody and incubated overnight at room temperature. The plate was then blocked by adding 150 µl of Reagent Diluent to each well and incubated for 1 hr at room temperature. HAEC total protein lysates were routinely pre-diluted 1/20 in PBS containing 25% FCS (within the linear range for the ELISA – Figure S1A), whilst conditioned media samples were undiluted. All samples and TM standards were subsequently added to the ELISA plate in duplicate at 50 µl/well. Assays proceeded for 2 hr at room temperature. The standard curve ranged from 31.25 to 2000 pg/ml of recombinant human TM (Figure S1B). Following sample incubation, 50 µl of the detection antibody was added to each well and then incubated for a further 2 hr at room temperature. Post-incubation, 50 µl of streptavidin-HRP was dispensed to each well and incubated for 20 min at room temperature in the dark. 50 µl of substrate solution was then added to each well and incubated for a further 20 min at room temperature in the dark. Reactions were terminated with the addition of 25 µl of stop solution to each well and the plate luminescence subsequently read at both 570 nm and 450 nm (wave correction was used to subtract the readings at 570 nm from 450 nm to correct for optical imperfections in the plate). For normalization purposes, TM levels in HAEC lysates were routinely presented as pg/µg of total protein, whilst TM levels in conditioned media were routinely presented as pg/10^5^ cells.

### Statistical analysis

Results are expressed as mean±s.e.m. Experimental points were performed in triplicate with a minimum of three independent experiments (n = 3). Statistical comparisons between control and experimental groups was by ANOVA in conjunction with a Dunnett's *post-hoc* test for multiple comparisons (*). A Student's *t*-test was also employed for pairwise comparisons (**α**, **δ**). A value of *P*≤0.05 was considered significant.

## Results

### Cyclic strain and shear stress modulate TM expression and release in HAECs

**Figure 1:**
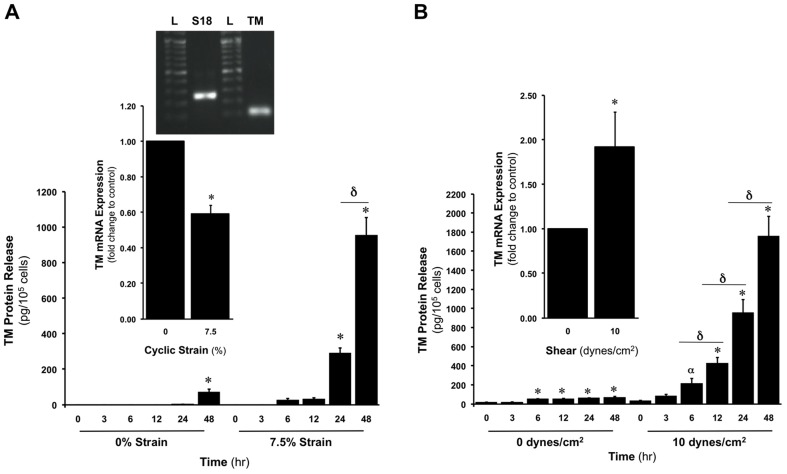
Effect of CS and LSS on TM expression and release in HAECs. (**A**) Time-dependent effect of CS (0 or 7.5%, 0–48 hr) on TM release. Inset shows effect of CS (0 or 7.5%, 24 hr) on TM mRNA levels. Agarose gel inset confirms predicted S18 and TM cDNA fragment sizes (250 and 107 bp, respectively) for designed primers. Gel is representative. Key: L, ladder. (**B**) Time-dependent effect of LSS (0 or 10 dynes/cm^2^, 0–48 hr) on TM release. Inset shows effect of LSS (0 or 10 dynes/cm^2^, 24 hr) on TM mRNA levels. *P≤0.05 versus unstrained or unsheared control. **α** *P* = 0.0008 versus 0 hr sheared control. **δ** *P*≤0.05.

The effect of CS (0 or 7.5%, 24 hr) on TM expression in HAECs was initially monitored. Relative to unstrained cells, we observed a significant reduction in TM mRNA (41%) in response to 7.5% strain (Figure 1A inset). The time-dependent release of TM from HAECs at 7.5% CS was also monitored over 48 hr, with significant levels of TM release apparent between 12–24 hr following strain onset (Figure 1). Importantly, no significant increase in apoptosis or loss in cell viability was observed in response to elevated strains (up to 12.5%), as monitored by flow cytometry using an Alexa Fluor 488 Annexin V-PI/Dead Cell Apoptosis Kit (Bio-Sciences)[28] and trypan blue exclusion assay, respectively (data not shown).

We next monitored the effect of LSS (0 or 10 dynes/cm^2^, 24 hr) on TM expression in HAECs. Relative to unsheared cells, we observed a significant increase in TM mRNA (92%) in response to 10 dynes/cm^2^ LSS (Figure 1B inset). The time-dependent release of TM from HAECs at 10 dynes/cm^2^ was also monitored over 48 hr. We observed that significant levels of TM release commenced between 6–12 hr following shear onset, with levels of released TM after 48 hr approximately 2-fold higher compared to CS-induced release (Figure 1B).

Considering the noticeable scarcity of published information on TM regulation by CS, it was therefore decided to focus exclusively on this stimulus for all subsequent experiments.

### CS downregulates TM protein expression

**Figure 2:**
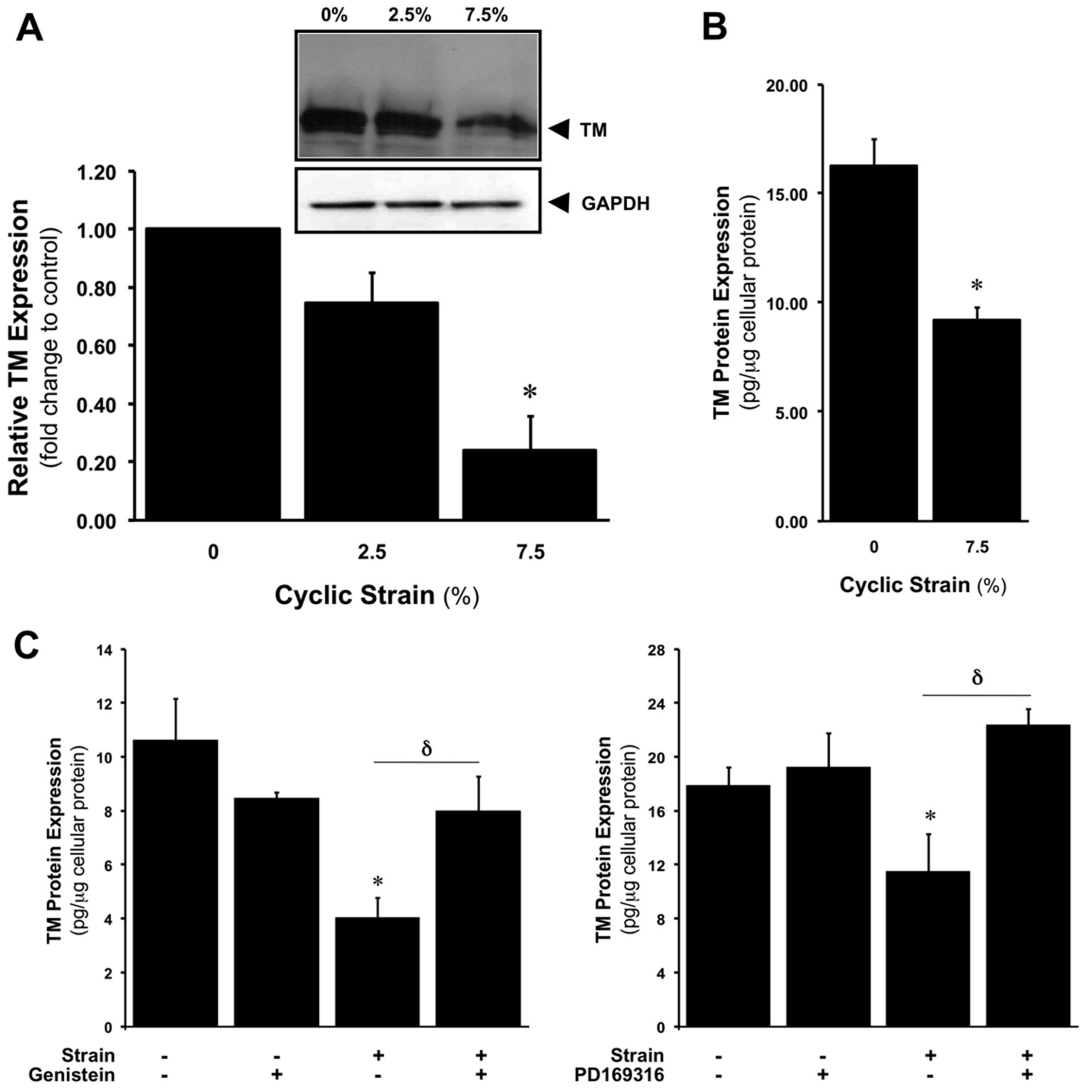
CS-dependent downregulation of TM protein expression in HAECs. Effect of CS (0–7.5%, 24 hr) on TM protein levels as monitored by (**A**) Western blotting and (**B**) ELISA. Histogram below gels represents the densitometric fold change in relative TM protein expression. *P≤0.05 versus 0% CS. All gels are representative. (**C**) Effect of 2 µM genistein and 10 µM PD169316 on CS-dependent downregulation of TM protein expression. *P≤0.05 versus untreated static control. **δ** *P*≤0.05.

Following strain experiments, total protein lysates were harvested and analysed for TM levels. Using Western blotting, CS (0–7.5%, 24 hr) was shown to reduce TM cellular protein levels in HAECs in a dose-dependent manner (Figure 2A). CS-dependent reduction in protein expression (∼43.5% at 7.5% CS for 24 hr) was also confirmed by ELISA (Figure 2B). In separate experiments, blockade of either receptor tyrosine kinase or p38 MAP kinase activation using genistein and PD169316, respectively, prevented the strain-dependent reduction in TM protein levels (Figure 2C).

### CS upregulates TM protein release

**Figure 3:**
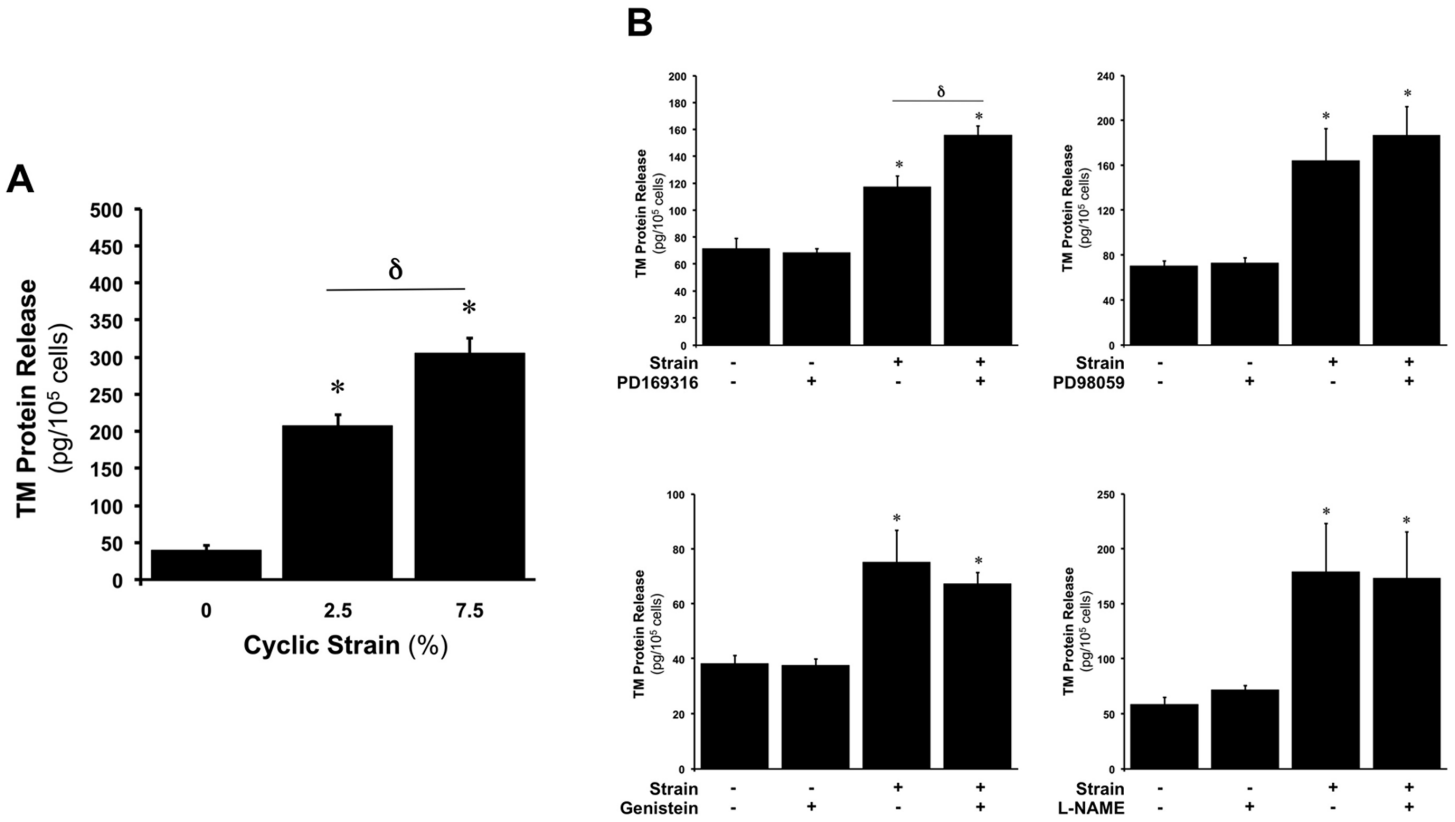
CS-dependent upregulation of TM protein release in HAECs. (**A**) Effect of CS (0–7.5%, 24 hr) on TM protein release. *P≤0.05 versus static control. **δ** *P*≤0.05. (**B**) Effect of 10 µM PD169316, 10 µM PD98059, 2 µM genistein and 1 mM L-NAME on CS-dependent upregulation of TM protein release. *P≤0.05 versus untreated static control. **δ** *P*≤0.05.

Following strain experiments, conditioned media was also harvested and analysed for TM levels by ELISA. In this regard, we observed that CS (0–7.5%, 24 hr) increased the release of TM into the media in a dose-dependent manner (Figure 3A), with maximal release occurring by 7.5% CS. In separate experiments, blockade of either receptor tyrosine kinase, MAP kinase (p38, ERK-1/2), or eNOS activation using genistein, PD169316/PD98059, and L-NG-nitroarginine methyl ester (L-NAME), respectively, did not attenuate the strain-dependent upregulation in TM release (Figure 3B).

### CS modulates TM expression and release in a frequency-dependent manner

**Figure 4:**
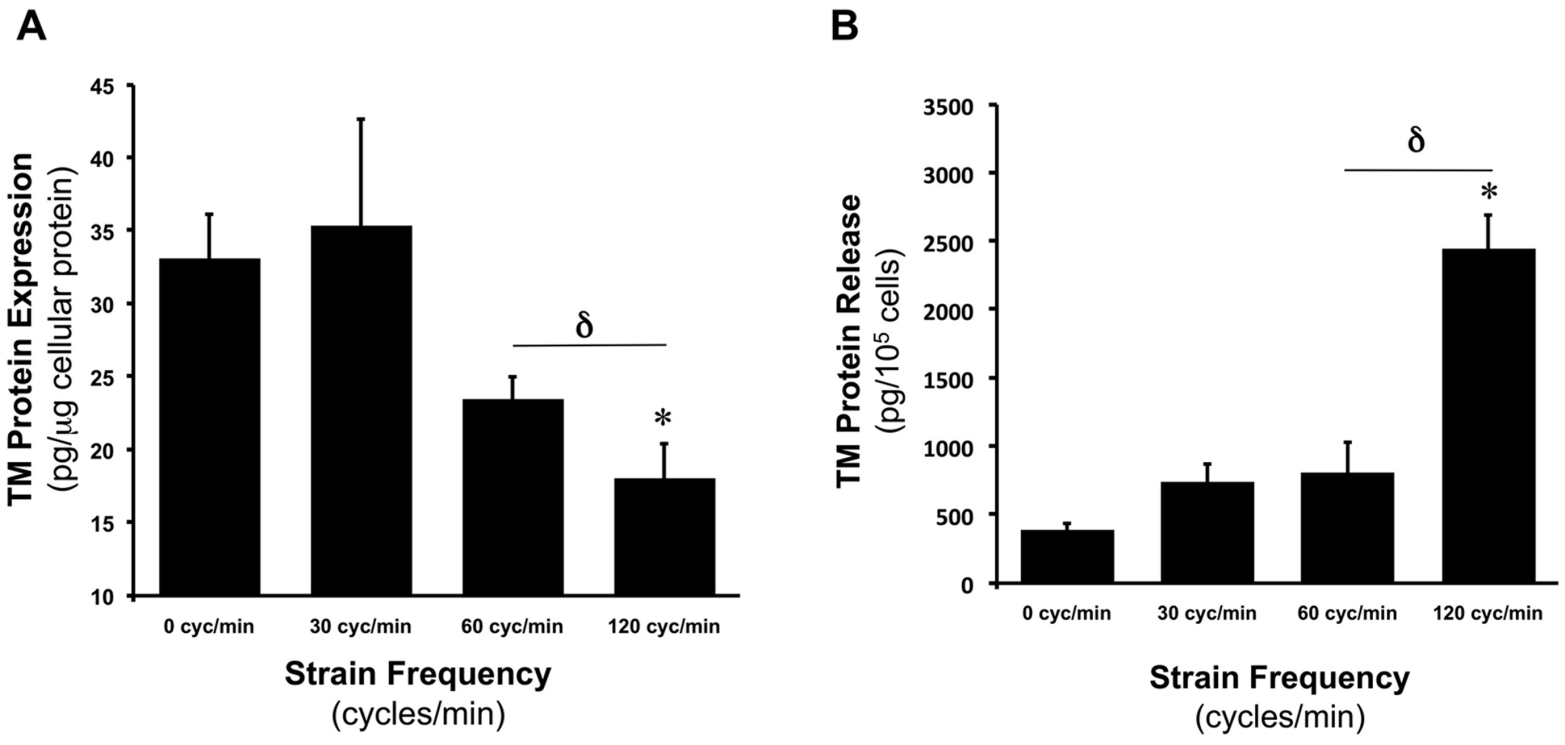
Frequency-dependent effects of CS on TM expression and release in HAECs. Effect of CS frequency (0% at 0 cycles/min versus 7.5% at 30–120 cycles/min for 24 hr) on (**A**) TM cellular protein levels and (**B**) TM release. *P≤0.05 versus static control. **δ** *P*≤0.05.

In another series of experiments, HAECs were subjected to a constant CS amplitude of 7.5% (24 hr), whilst frequency was varied (0.5, 1, and 2 Hz – corresponding to 30, 60 and 120 cycles per min). Following strain experiments, cell lysates and conditioned media were harvested for analysis of TM levels by ELISA. Relative to 0% (0 Hz) controls, increasing CS frequency progressively decreased TM protein expression, with lowest TM levels observed at 2 Hz (Figure 4A). Analysis of conditioned media however, revealed a profound increase in TM release (6.3-fold) when strain frequency was increased from 0 to 2 Hz (Figure 4B).

### CS-induced TM release is enhanced in the presence of inflammatory mediators

**Figure 5:**
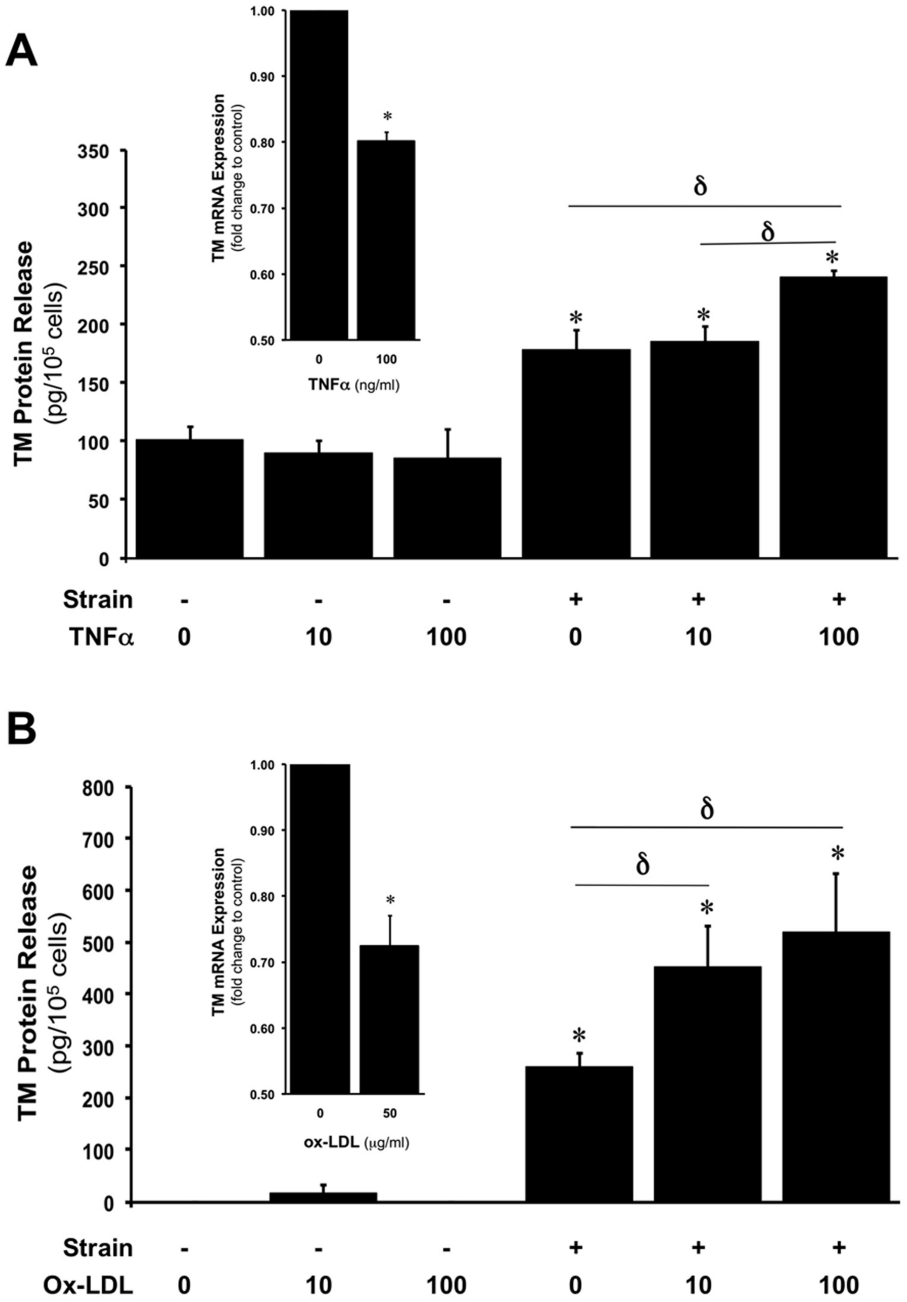
Effects of inflammatory mediators on CS-induced TM release in HAECs. (**A**) Effect of CS (0 or 7.5%, 24 hr) on TM release from HAECs in the presence of 0, 10, and 100 ng/ml of TNFα. (**B**) Effect of CS (0 or 7.5%, 24 hr) on TM release from HAECs in the presence of 0, 10, and 50 µg/ml of ox-LDL. *P≤0.05 versus untreated 0% CS. **δ** *P*≤0.05. Histogram insets in (**A**) and (**B**) show effect of upper concentrations of TNFα and ox-LDL, respectively, on TM mRNA levels. *P≤0.05 versus untreated controls.

HAECs were subjected to CS (0 or 7.5%, 24 hr) in the absence and presence of tumour necrosis factor-α (TNFα, 0–100 ng/ml) and oxidised low density lipoprotein (ox-LDL, 0–50 µg/ml), concentration ranges consistent with those previously employed for these agents in other *in vitro* cell studies [28], [29]. In static HAECs, neither reagent was seen to significantly induce TM release. By contrast, in strained HAECs, elevated concentrations of either TNFα (100 ng/ml, Figure 5A) or ox-LDL (10–50 µg/ml, Figure 5B) led to significantly higher levels of TM release relative to strained untreated HAECs. Additionally, static HAEC treatment for 24 hr with TNFα (100 ng/ml) and ox-LDL (50 µg/ml) downregulated TM mRNA levels by 20% and 28%, respectively (Figure 5A,B insets). By contrast, elevated glucose (15–30 mM) had no significant effect on TM expression levels.

### CS-induced TM release from HAECs is protease-independent and microparticle-dependent

**Figure 6:**
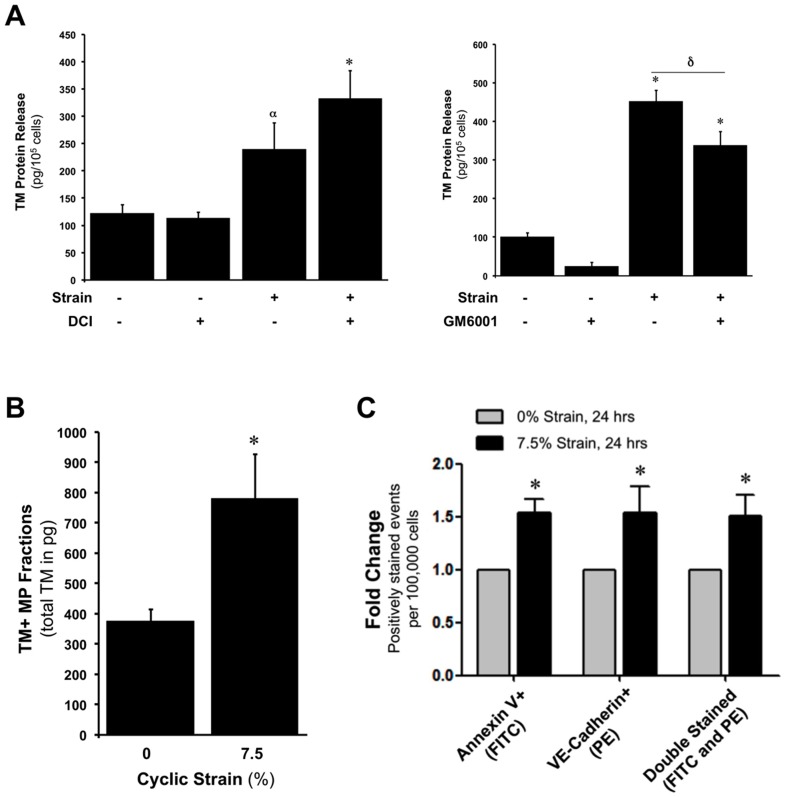
Proteolytic and non-proteolytic mechanisms of TM release from HAECs. (**A**) Effect of protease inhibitors, DCI (rhomboids) and GM6001 (MMPs), on TM release from HAECs under static and strain (0 or 7.5%, 24 hr) conditions. (**B**) Confirmation of TM presence in HAEC microparticle (MP) fractions harvested from conditioned media following CS treatment (0 or 7.5% strain, 24 hr). (**C**) Confirmation of CS-dependent increase in annexin V+/VE-Cadherin+ HAEC MP release following CS treatment (0 or 7.5% strain, 24 hr). *P≤0.05 versus untreated 0% CS. **α** *P* = 0.045 versus untreated 0% CS. **δ** *P*≤0.05.

HAECs were subjected to CS (0 or 7.5%, 24 hr) in the absence and presence of 10 mM GM6001 or 10 mM 3,2-dichloroisocoumarin (DCI), selective inhibitors of matrix metalloproteinases (MMPs) and rhomboids [30], [31], respectively, proteases which have been previously implicated in the release of TM from cells [32], [33]. In this respect, neither inhibitor was found to significantly block this effect (following correction for any baseline inhibitor effects)(Figure 6A). In a final series of experiments, HAECs were again subjected to CS (0 or 7.5%, 24 hr) and the conditioned media harvested for extraction of microparticles by high speed centrifugation. CS was found to upregulate the release of annexin V+/VE-cadherin+ MPs by 1.5 fold (Figure 6C). Lysed microparticle fractions were subsequently analysed for TM levels by ELISA. Microparticle extracts from both static and strained HAECs were found to be positive for TM, with strained fractions consistently exhibiting up to 2.1 fold more TM than static fractions (Figure 6B).

## Discussion

TM is a pivotal determinant of endothelial thromboresistance and vessel homeostasis. TM-mediated binding of thrombin leading to protein C activation has beneficial anti-coagulant, anti-fibrinolytic, and anti-inflammatory outcomes for the vessel wall. In the current paper we employed *in vitro* HAEC culture models to investigate the regulatory influence of physiologic hemodynamic forces, namely, equibiaxial cyclic strain and laminar shear stress, on endothelial TM expression and release, with particular emphasis placed on the former stimulus. Our initial investigations clearly demonstrated that cyclic strain could significantly downregulate TM expression (mRNA and protein), an event which was found to be sensitive to inhibition of both p38 MAPK and receptor tyrosine kinase signaling, and which contrasts sharply with the observed upregulatory effect of shear stress on TM expression. These observations are consistent with previous reports into the apparently *opposite* effects of cyclic strain [18] and shear stress [14]–[17] on vascular endothelial TM expression, and highlight the likelihood that basal levels of endothelial TM expression under physiological conditions *in vivo* most likely represent a careful balance between a myriad of potentially opposing stimuli [1]. As such, downregulatory stimuli (e.g. cyclic strain, cAMP, c-reactive protein, phorbolesters) would likely balance out the effects of upregulatory stimuli (e.g. shear stress, thrombin, VEGF), helping to establish a steady state TM expression level under normal physiological conditions. By way of a possible transcriptional explanation for the opposite effects of strain and shear on TM expression, the 5′untranslated region of the TM promoter displays a *CACCC* motif, which is known to facilitate positive gene regulation by Krüppel-like factor 2 (KLF2), a transcription factor that is activated by shear stress, but not by cyclic strain [34], [35]. The cyclic strain-dependent activation of NF-κB [36], an established negative regulator of TM expression [37] is also of relevance. Finally, it can be noted that our results differ from those of Chen *et al.* who demonstrate that 18 hr exposure of HUVECs to 21% (but not 15%) cyclic strain leads to induction of TM expression (>2-fold), an increase which the authors attribute to NO-mediated stabilization of TM protein via S-nitrosylation, and not to transcriptional activation [19]. Differences in cell type (HAEC versus HUVEC), applied strain level (physiological versus pathological), and strain system (FX4000T versus FX2000) may all account for the observed disparity between these observations.

Interestingly, there have been no *in vitro* studies thus far comprehensively documenting the influence of either hemodynamic force on endothelial TM release. In this respect, our next investigations demonstrated time-dependent release of TM from HAECs in response to physiologic levels of cyclic strain and shear stress. With continuing focus on the former stimulus, our studies subsequently demonstrated that physiologic cyclic strain could induce TM release from HAECs in both a dose- and frequency-dependent manner. Moreover, we noted that the cyclic strain-induced release of TM was not effected by inhibition of either MAP kinase (p38, ERK1/2), receptor tyrosine kinase, or endothelial nitric oxide synthase (eNOS) activation. The release or shedding of TM from cell surfaces has received much attention in the literature [1]. As circulating TM levels in plasma are typically in the ng/ml range in healthy humans, this would suggest that the stimulated release of TM by physiologic hemodynamic forces may contribute to normal vessel homeostasis. In this respect, it is worth noting that circulating TM has been shown to bind thrombin and exhibit anti-coagulant ability [38].

The potent increase in TM release (∼3-fold) that accompanied a doubling of the strain frequency (from 1 to 2 Hz) is also quite noteworthy. The observed frequency-dependent induction of TM release may involve a cytoskeletal mechanism, given the pivotal role of the actin cytoskeleton in cellular secretory and microvesiculation processes [39], [40]. In support of this, recent work by Hsu *et al.* has shown that endothelial stress fibre alignment and turnover are highly sensitive to strain frequency [41]. The increase in strain frequency is also analogous to an elevation in normal heart rate (i.e. from 60–120 beats per minute), and in this respect it can be noted that increased levels of circulating TM have been associated with physical exercise [42], [43], which is characterised by elevated shearing and heart rate.

As endothelial activation *in vivo* may involve the combined influence of both hemodynamic *and* humoral stimuli, we next decided to examine the concerted impact of cyclic strain and inflammatory mediators, namely TNFα and ox-LDL, on TM release from HAECs (in associated studies, the effect of elevated glucose levels, ranging from normal to diabetic, was also examined – Figure S2). Our investigations initially confirmed downregulation of TM mRNA levels in response to TNFα treatment of HAECs. This is consistent with other studies [44], [45] and likely reflects the proinflammatory nature of TNFα. We also noted that TNFα treatment alone (0–100 ng/ml) had no significant effect on TM release from unstrained HAECs. This is consistent with similar observations by Boehme *et al.* who reported that TNFα treatment alone had no effect on TM release from HUVECs [46]. Interestingly however, these authors (and others) noted that endothelial TM release was significantly increased following the concerted action of TNFα and neutrophils, likely arising from cytokine activation of neutrophil elastase and cathepsin G, which may cause enzymatic release of TM from endothelial cells [46]–[48]. We further noted that 100 ng/ml TNFα combined with 7.5% strain appeared to potentiate TM release from HAECs. The reason for this is as yet unknown. In this respect, it is noteworthy that the ROS-generating ability of cyclic strain in vascular endothelial cells has previously been found to be significantly potentiated by co-treatment with TNFα [49], leading us to speculate that a ROS-dependent mechanism may account for our observations. This appears unlikely however given the potent ROS-induction that would have accompanied the treatment of endothelial cells with TNFα alone [28]. Finally, the possibility that TNFα treatment may be enhancing TM internalization via endocytosis, thereby reducing surface levels of TM available for release either by enzymatic or microparticle means, is also a potential mechanistic consideration under static and strain conditions [50], [51]. Whilst beyond the scope of the present study, these mechanistic concepts surrounding TM release merit further investigation.

In related experiments, it was noted that ox-LDL could downregulate TM mRNA levels in HAECs, again consistent with earlier studies [29]. As with TNFα, it was also noted that ox-LDL treatment alone (0–50 µg/ml) had no significant effect on TM release from unstrained HAECs, whilst appearing to potentiate TM release under 7.5% strain at the elevated ox-LDL concentrations (10–50 µg/ml). It has previously been demonstrated that the concerted action of cyclic strain and ox-LDL can synergistically increase the expression of lectin-like ox-LDL receptor-1 (LOX-1), albeit in chondrocytes and vascular smooth muscle cells [52], [53], with consequences for cell signalling and functions. Whilst undetermined at this time, this phenomenon may also be relevant to the concerted action of ox-LDL and cyclic strain on TM release from HAECs. It is also noteworthy that ox-LDL can attenuate agonist-stimulated nitric oxide (NO) release from HUVECs [54]. Given the regulatory cross-talk that exists between thrombomodulin and NO [55], blockade of strain-dependent eNOS activation and NO release by ox-LDL may constitute another possible mechanism for its potentiation of TM release. Finally, it can be noted that clinical studies have demonstrated a direct correlation between circulating ox-LDL and sTM levels [56], [57].

In a final series of experiments, we sought to better understand the mechanism of TM release from HAECs in response to cyclic strain. A considerable volume of research has previously focused on the proteolytic release of TM from cells into tissue fluids such as plasma, urine, vitreous fluid, and synovial fluid [1]. In this regard, our investigations did not indicate a significant role for either MMPs or rhomboids (RHBDL2-like intramembrane serine proteases) in the strain-induced release of TM from HAECs, despite previous studies linking these enzymes to sTM shedding in endothelial and non-endothelial cells, respectively [32], [33]. In addition to proteolytic release however, a few studies point to the microvesicular pathway (microparticles and/or exosomes) as a mechanism for TM shedding from activated cells [58], prompting us to investigate this possibility in our HAEC cyclic strain model. Following preparation of microparticle fractions from HAEC-conditioned media by high speed centrifugation [24], [25], analysis revealed significantly higher quantities of TM in microparticle fractions from strained cells, suggesting that physiologic cyclic strain can stimulate release of TM(+) microparticles from HAECs. Although speculative at present, strain-induced TM(+) endothelial microparticles could exhibit anti-coagulant and anti-inflammatory effects to help fine-tune vascular homeostasis. This is consistent with the general view that endothelial microparticles function as important conveyors of biological information capable of biomolecule dissemination and exchange with other vascular cells. Whilst frequently viewed as being indicative of endothelial activation and apoptosis, and of reflecting potentially deletarious consequences for vascular homeostasis and disease progression, more recent studies have demonstrated the *plasticity* of endothelial microparticles with respect to phenotype and function. Indeed, several studies have now confirmed the ability of endothelial microparticles to promote cytoprotective effects and endothelial repair, as well as exhibiting anti-coagulant and anti-inflammatory actions [59]. Finally, in light of our observations, it is also noteworthy that both cyclic strain and shear stress have very recently been demonstrated to regulate microparticle generation in cultured endothelial cells [60], [61].

In summary, an investigation into the effects of hemodynamic forces, and specifically cyclic strain, on TM regulation in HAECs is presented. Physiologic cyclic strain was observed to downregulate TM expression (mRNA and protein), whilst upregulating in a time-, dose- and frequency-dependent manner the release of TM into media. Inflammatory mediators, TNFα and ox-LDL, were observed to potentiate strain-induced TM release. Moreover, evidence is also presented in support of a microvesicular mechanism for TM release by cyclic strain. To our knowledge, these are the first *in vitro* studies to comprehensively assess the regulatory influence of physiologic cyclic strain on vascular endothelial TM dynamics. It should be noted however that these are preliminary investigations using vascular cell cultures and model systems (e.g. Flexercell) that, whilst well established within the literature, only offer a partial approximation of *in vivo* physiology. As such, follow up studies using more advanced *ex vivo* and *in vivo* models of hemodynamic loading will ultimately be necessary to reinforce the physiological conclusions made in our study.

## Supporting Information

Figure S1 **Performance characteristics for the human thrombomodulin/BDCA-3 DuoSet ELISA.** (**A**) Linear range of the ELISA monitored over a broad range of HAEC lysate concentrations (Note: cell lysates were routinely assayed in the 1∶20 dilution range). (**B**) ELISA standard curve (0–2000 pg/ml).
(TIF)

Figure S2 **Effects of elevated glucose on CS-induced TM release in HAECs.** Effect of CS (0 or 7.5%, 24 hr) on TM release from HAECs in the presence of 5, 15, and 30 mM glucose. *P≤0.05 versus 5 mM 0% CS. **δ** *P*≤0.05 versus 5 mM 7.5% CS.
(TIF)
