# Supplementary figures and images for: Regulation of Thrombomodulin Expression and Release in Human Aortic Endothelial Cells by Cyclic Strain
Source: PLoS One. 2014 Sep 19;9(9):e108254. doi: 10.1371/journal.pone.0108254 (PMC4169621; doi:10.1371/journal.pone.0108254)

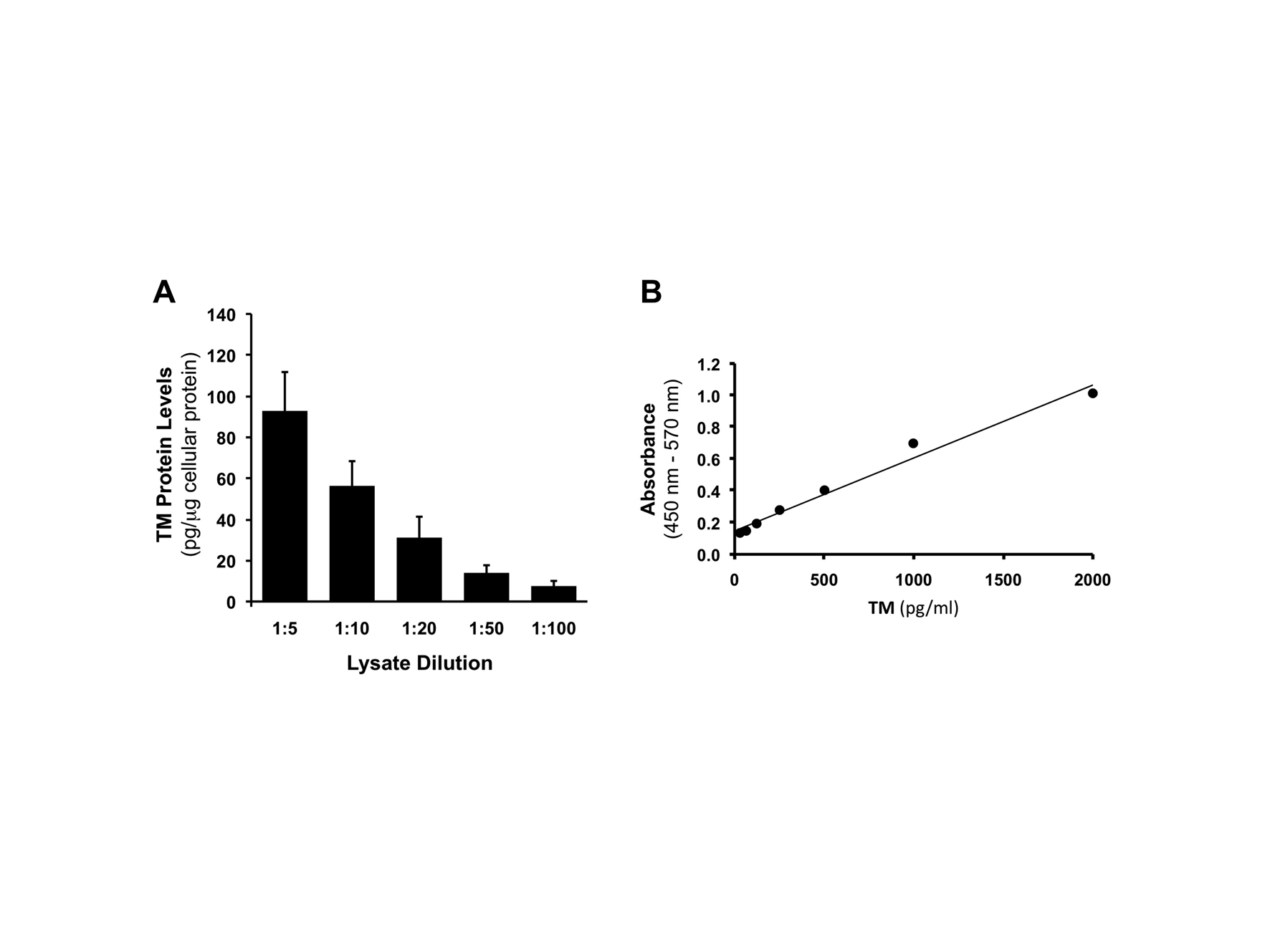

Supplement: Figure S1 — Performance characteristics for the human thrombomodulin/BDCA-3 DuoSet ELISA. (A) Linear range of the ELISA monitored over a broad range of HAEC lysate concentrations (Note: cell lysates were routinely assayed in the 1∶20 dilution range). (B) ELISA standard curve (0–2000 pg/ml). (TIF) [file pone.0108254.s001.tif]

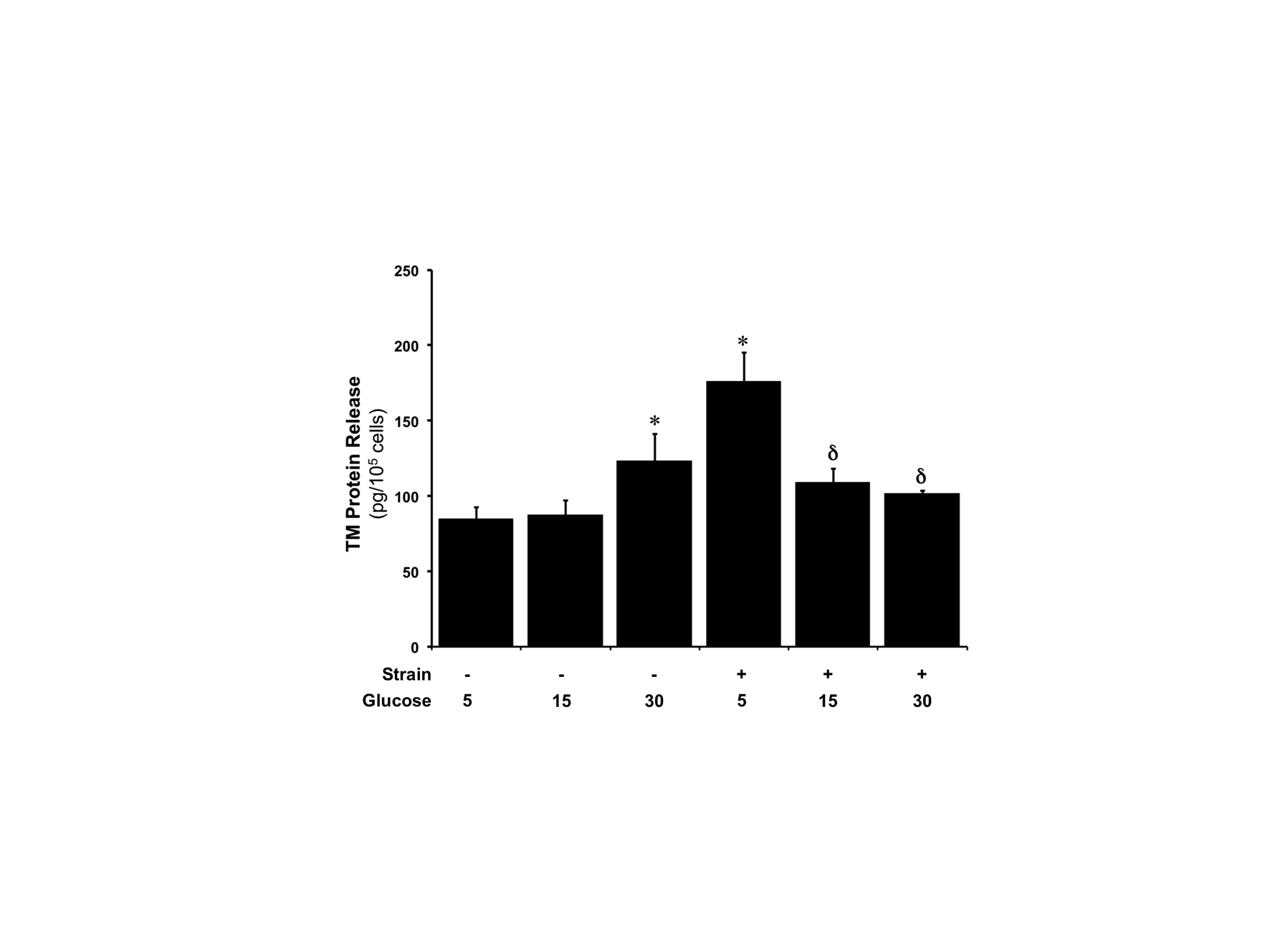

Supplement: Figure S2 — Effects of elevated glucose on CS-induced TM release in HAECs. Effect of CS (0 or 7.5%, 24 hr) on TM release from HAECs in the presence of 5, 15, and 30 mM glucose. *P≤0.05 versus 5 mM 0% CS. δ P≤0.05 versus 5 mM 7.5% CS. (TIF) [file pone.0108254.s002.tif]
